# Supplementary material for: Cell-Death Metabolites from Cocconeis scutellum var. parva Identified by Integrating Bioactivity-Based Fractionation and Non-Targeted Metabolomic Approaches
Source: Mar Drugs. 2024 Jul 18;22(7):320. doi: 10.3390/md22070320 (PMC11278434; doi:10.3390/md22070320)
Supplement: Supplementary file 1 [file marinedrugs-22-00320-s001.zip › marinedrugs-3039331-supplementary.pdf]

marine drugs

Supplementary material

Cell-death metabolites from *Cocconeis scutellum parva* identified by integrating bioactivity-based fractionation and non-targeted metabolomic approaches.

Carlos Sanchez-Arcos [1], Mirko Mutalipassi [2,3], Valerio Zupo [4], Eric von Elert [1*]

[1] Institute for Zoology, Cologne Biocenter University of Cologne, 50674 Köln, Germany
[2] Department of Integrative Marine Ecology, Stazione Zoologica Anton Dohrn, 80122 Napoli, Italy
[3] NBFC, National Biodiversity Future Center, 90133 Palermo, Italy
[4] Integrative Marine Ecology Department. Stazione Zoologica Anton Dohrn. Ischia Marine Center. Punta San Pietro. 80077 Ischia (Italy)
* Correspondence: evelert@uni-koeln.de (EvE); Tel.: +49-221-470-6084 (EvE)

Citation: To be added by editorial staff during production.

Academic Editor: Firstname Lastname

Received: date
Revised: date
Accepted: date
Published: date

Copyright: © 2024 by the authors. Submitted for possible open access publication under the terms and conditions of the Creative Commons Attribution (CC BY) license (https://creativecommons.org/licenses/by/4.0/).

Supplementary figures

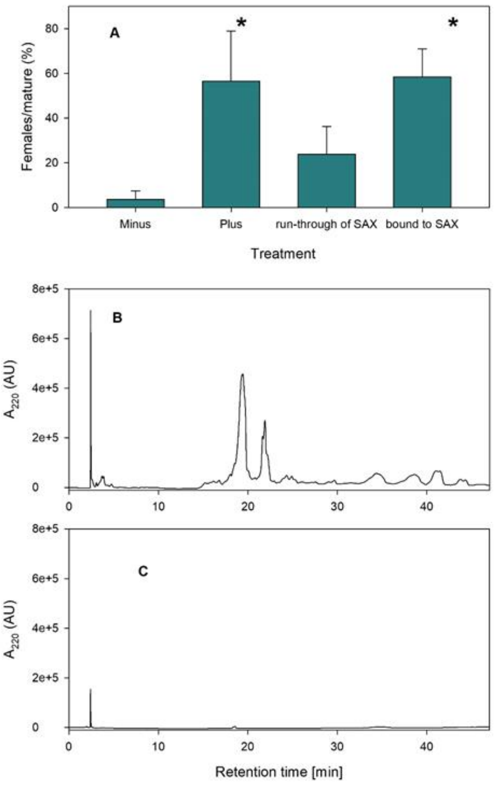

Figure S1: Effects of strong anion exchanger on Cocconeis scutellum var. parva extract. A) Morphogenetic activity of *C. scutellum* var. *parva* extract expressed as a percentage of females relative to the total number of mature animals. Depicted are mean +/- SE activity of n = 3 biological replicates of of negative ('Minus'), positive control ('Plus'), that part of *C. scutellum parva* extract which had not been retained by strong anion exchanger ('run through of SAX') and that part which had been retained ('bound to SAX'). * indicate significant differences to 'Minus after z-test. B) HPLC chromatogram of *C. scutellum* var. *parva* extract without SAX purification and C) after binding to SAX and subsequent elution from SAX. The chromatograms have been recorded at 220 nm.

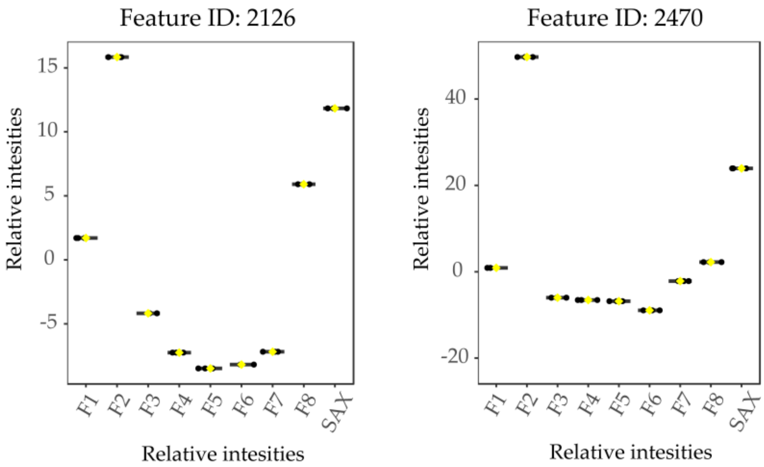

Figure S2. Relative levels of features ID 2126 and ID 2470 among all the active fractions and SAX extract from Table 1.

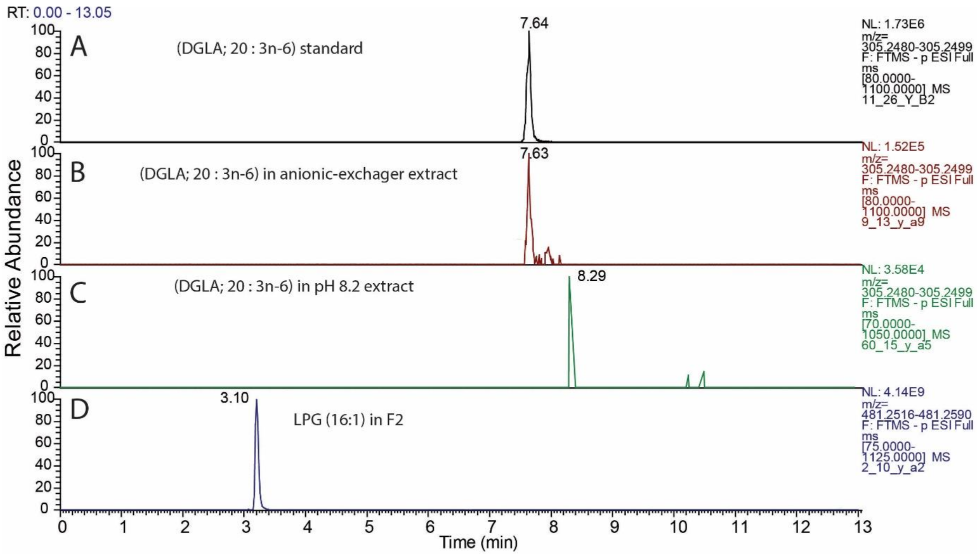

Figure S3. Extracted-ion chromatograms of DGLA (20:3n-6) m/z 305.2458 in A) DGLA standard, in B) in *C. scutellum* var. *parva* anionic-exchanger extract, C) in *C. scutellum* var. *parva* pH 8.2 extract. D) Extracted-ion chromatograms of LPG (16:1) m/z 481.2570 in *C. scutellum* var. *parva* active fraction F2.
